# COVID-19: an opportunity for African governments to rethink social welfare benefits and protection

**DOI:** 10.11604/pamj.supp.2020.35.2.23875

**Published:** 2020-06-05

**Authors:** Ikenna Desmond Ebuenyi

**Affiliations:** 1 Assisting Living & Learning (ALL) Institute, Department of Psychology, Maynooth University, Maynooth, Ireland

**Keywords:** COVID-19, African governments, social welfare benefits, social protection

## Abstract

The emergence of COVID-19 in December 2019 has highlighted several lessons about Public health emergencies. One important lesson is on the role of social welfare benefits and protection in the overall management of public health emergencies. The absence of a functional and digitalized social welfare system in Africa may render ineffective public health measures to mitigate the spread of COVID-19. The social determinant of disease illustrates the nexus between poverty and health outcomes. Therefore, COVID-19 is an opportunity for African governments to rethink their stance on social welfare benefits and protection; and adopt mechanisms that protect the most vulnerable.

## Commentary

The emergence of COVID-19 in December 2019 has severely affected work and employment globally. According to the International Labour Organisation (ILO), about 25 million jobs may be lost worldwide as a result of COVID-19 [1]. The predicted increase in extreme poverty led to ILO´s proposed policy framework to cushion the effect of COVID-19 on the lives of people everywhere and especially vulnerable populations [2] Extending Social protection to everyone was one of the measures proffered by the ILO to address the impact of the pandemic on work; especially for those engaged in the informal economy [1].

This recommendation is commendable and very essential on account of widespread socio-economic impact of COVID-19 globally. In the United States of America, the Coronavirus Aid, Relief, and Economic Security Act (CARES Act) provided more than $2 trillion to support families and small businesses affected by the Covid-19 pandemic. In Ireland, the government launched the COVID-19 Pandemic Unemployment Payment which is a new social welfare payment of €350 a week; in addition to other measures to support enterprises. In Japan, the government in response to the socio-economic impact of COVID-19 on the people commenced a cash payment of **¥**100k each to all residents in Japan [2]. In Africa, 47 (87.0%) countries have responded with various social protection measures to reduce the impact of COVID-19 on their citizens [3]. As of 25 May 2020, 188 countries and territories had either announced or implemented diverse social protection measures to mitigate spread and impact of COVID -19. However, anecdotal evidence and opinions suggest there may be a difference between announced policies and its implementation especially in sub Saharan Africa [4,5].

According to Rutkowsk and colleagues, Government to person (G2P) payments are very pivotal in reducing the socio-economic consequences of COVID-19. However, they suggested that the success of this may be dependent on countries with pre-existing and advanced G2P payment systems that is digitalized and able to reach people when they need it [6]. Studies indicate that in Africa and most low-income settings, social welfare systems are still not common [4,7]. A vast majority of persons in need of social welfare are still dependent on their families and community for help. Unfortunately, the capacity of the family and community to fill this pivotal role of the government has been eroded by the COVID -19 pandemic which affected the ‘needy and their helpers’. Some of the measures (e.g. lockdown) adopted to mitigate the effect of the pandemic led to job/income loss globally, and particularly in Africa with a preponderance of the informal economy. The absence of social welfare and work support in the informal economy makes it very difficult for workers in Africa to endure job loss and also adhere to recommended measures to mitigate the effect of COVID -19 [1]. Social protection is a useful tool for protecting disadvantages families and vulnerable populations in Africa. However, just like in the Inverse-care law which suggest that available healthcare varies inversely with the population [8], access to social protection appear to exist more in countries where people had social protection before the advent of COVID-19.

This deficiency in social welfare and protection may lead to unintended health and socio-economic consequences. The social distancing and stay at home measures by governments, may work in climes where technology exist to work from home and where employers are supported by the government to provide needed accommodations for employees. However, in informal work settings where people need to go out for daily pay and where no cash transfer is coming to the people, it is common for people to ignore lockdown advice and the health consequences to fulfil immediate challenges of hunger. It is common to ignore poverty, while at the same time investing in health development. However, the important lessons from the social determinants of disease indicates that we cannot ignore the impact poverty on health [9]. Socioeconomic disadvantages heighten vulnerability to health and social risk. Social protection is considered relevant in the response and management of public health emergencies [5,6]. Hence, the absence of an effective and ‘real’ social welfare system may worsen the effect of COVID-19 in Africa.

Therefore, COVID-19 presents an opportunity for African governments to rethink social welfare benefits and protection. The importance of a social welfare system in Africa cannot be over emphasized and the need for a robust and digitalized systems for G2P payments have been highlighted by the COVID -19 pandemic. Digitalized social welfare payment systems are cheaper than ineffective subjective manual payments adopted by most African states which often do not guarantee that recipients receive the transfers [6,10]. The time to democratise social welfare is now and African governments must ensure that it reaches those that need it and plans made for its extension to all in times of crisis. It is time for African governments to mainstream social welfare because in times of ‘crisis’, almost everyone may need a helping hand.

## Competing interests

The author declares no competing interests.
